# Factors for Predicting Favorable Outcome of Percutaneous Epidural Adhesiolysis for Lumbar Disc Herniation

**DOI:** 10.1155/2017/1494538

**Published:** 2017-01-26

**Authors:** Sang Ho Moon, Jae Il Lee, Hyun Seok Cho, Jin Woo Shin, Won Uk Koh

**Affiliations:** ^1^Department of Orthopedic Surgery, Seoul Sacred Heart General Hospital, Wangsan-Ro 259, Dongdaemun-gu, Seoul 02488, Republic of Korea; ^2^Department of Anesthesiology and Pain Medicine, Asan Medical Center, University of Ulsan College of Medicine, 88 Olympic-ro 43 gil, Songpa-Gu, Seoul 05505, Republic of Korea

## Abstract

*Background*. Lower back pain is a common reason for disability and the most common cause is lumbar disc herniation. Percutaneous epidural adhesiolysis has been applied to relieve pain and increase the functional capacity of patients who present this condition.* Objectives*. In this study, we retrospectively evaluated the factors which predict the outcome of percutaneous epidural adhesiolysis in patients who were diagnosed with lumbar disc herniation.* Methods*. Electronic medical records of patients diagnosed with lumbar disc herniation who have received percutaneous epidural adhesiolysis treatment were reviewed. The primary outcome was the factors that were associated with substantial response of ≥4 points or ≥50% of pain relief in the numerical rating scale pain score 12 months after the treatment.* Results*. Multivariate logistic regression analysis demonstrated that the presence of high-intensity zone (HIZ) at magnetic resonance imaging was a predictor of substantial response to percutaneous epidural adhesiolysis for 12 months (*P* = 0.007). The presence of a condition involving the vertebral foramen was a predictor for unsuccessful response after 12 months (*P* = 0.02).* Discussion and Conclusion*. The presence of HIZ was a predictor of favorable long-term outcome after percutaneous epidural adhesiolysis for the treatment of lower back pain with radicular pain caused by lumbar disc herniation.

## 1. Introduction

Lower back pain is one of the most common causes of disability worldwide and is often accompanied by radicular pain [1, 2]. The most common cause of lumbar radicular pain is lumbar disc herniation, which is also the most common cause of lower back pain [3, 4]. Although the majority of patients who present with lower back pain and radicular pain improve within 12 weeks, between 6% and 11% of patients continue to have symptoms that persist for more than 3 months, which can lead to medical expenses and disability [3, 5].

Although the natural course in more than 80% and 90% of patients is symptom improvement within the first 6 and 12 weeks, respectively, treatment for relief during the symptomatic period is essential, as significant disability during daily activities may occur [6]. Initial treatment includes conservative management with oral medication for pain relief and physical therapy [7]. If the symptoms persist or the improvements are insufficient, interventions such as epidural steroid injections or, in limited cases, percutaneous epidural adhesiolysis can be applied [4, 8–10]. If the symptoms persist after these interventions, surgery should be considered [11–13].

Since its introduction in the late 1990s, percutaneous epidural adhesiolysis has been applied to relieve pain and increase the functional capacity of patients who present chronic lower back pain with or without radiculopathy, which are refractory to conservative treatment [14, 15]. Many studies have reported the effects of percutaneous epidural adhesiolysis in patients who present persistent symptoms of lower back pain with or without radicular pain diagnosed with various pathological conditions [16–18]. There is sufficient evidence of the short-term (<3 months) efficacy of percutaneous epidural adhesiolysis and moderate evidence on its mid- to long-term (>3 months) efficacy [19]. Indeed, the strength of evidence varies, depending on the diagnosed pathological condition. Two previous studies have described the effects of percutaneous epidural adhesiolysis on the treatment of lumbar disc herniation, and one study analyzed its effects on lumbar disc herniation and postlaminectomy syndrome [9, 10].

In this study, we retrospectively evaluated the factors predicting the outcome of percutaneous epidural adhesiolysis on patients diagnosed with lumbar disc herniation who present symptoms of lower back pain with radicular pain that were refractory to conservative treatments including epidural steroid injections.

## 2. Methods

This study was approved by the local ethics committee (BD2015-60), and the necessity to obtain informed consent was waived because of the retrospective nature of the analysis that reviewed previously recorded patient data. The electronic medical records of patients who were diagnosed with lumbar disc herniation presenting symptoms of lower back pain with radicular pain that have received percutaneous epidural adhesiolysis treatment were reviewed. The data for every patient treated with percutaneous epidural adhesiolysis for this condition at Seoul Sacred Heart General Hospital between November 2011 and October 2013 were collected. Inclusion criteria for the patient data collection included the following: patient age > 20; symptoms of lower back pain with radiculopathy; subacute or chronic symptoms lasting more than 6 weeks; failure to respond to conservative treatment including medication and physical therapy; symptoms refractory to interlaminar or transforaminal epidural injections; definitive diagnosis of lumbar disc herniation which was confirmed by magnetic resonance imaging (MRI). The percutaneous epidural adhesiolysis technique was standardized to all patients receiving the procedure. Each patient received a transforaminal epidural injection twice in 2- or 3-week intervals, and if the symptoms persisted or the relief was insufficient, the patient received percutaneous epidural adhesiolysis after a >1-month interval between the last epidural block. The patient data including any one of the following exclusion criteria were removed from the analysis: inadequate data or loss to follow-up before the first year after percutaneous epidural adhesiolysis, diagnosis other than lumbar disc herniation, history of previous percutaneous epidural adhesiolysis, previous lower back or lower limb surgery, and percutaneous epidural adhesiolysis techniques other than the catheter-guided technique.

### 2.1. Percutaneous Epidural Adhesiolysis Technique

**Figure 1:**
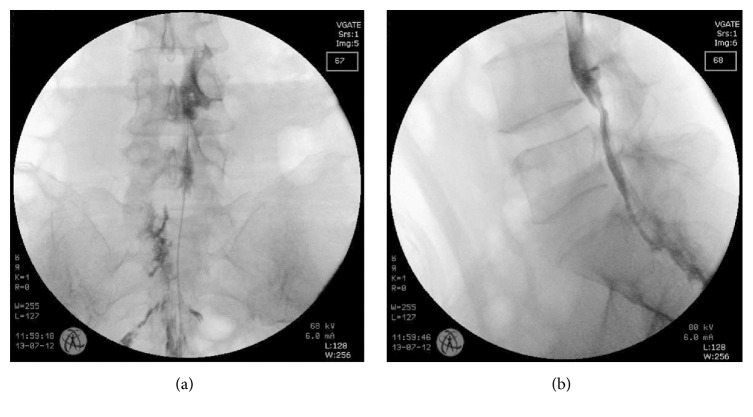
Fluoroscopic image of a patient receiving a percutaneous epidural adhesiolysis. Anteroposterior view (a) and lateral view (b).

All of the procedures were performed under fluoroscopic guidance on an outpatient basis. The patient was placed in a prone position with a pillow under the abdomen to secure the position and minimize lumbar lordosis. The administration of opioids or sedatives for mild sedation and analgesia during the procedure was decided at the discretion of the attending physician. The caudal approach was used under fluoroscopic guidance. The 16 G RK Tuohy introducer needle was inserted through the sacrococcygeal ligament and placed in the sacral canal. A radiopaque contrast dye (Ultravist, Bayer Korea, Seoul, Korea) was injected via the introducer needle to confirm epidural placement of the needle, and epidural filling defects were identified by the epidurogram. The filling defects were further compared with the MRI findings and the dominant symptom of the patient. After appropriate confirmation of the pathology, a metal-reinforced Racz epidural catheter (Epimed, Farmers Branch, TX) was inserted through the epidural needle towards the target area, which was determined by imaging and patient symptoms. A radiopaque contrast dye was again injected to confirm the appropriate position of the catheter tip at the site of pathology and to further verify any intravascular, intrathecal, or other extraepidural filling (Figure 1). The catheter tip was positioned at the anterior epidural space of the target site, and in the case of foraminal diseases, was placed at the lateral recess or opening of the foramen. After confirmation of the catheter tip position, 3–5 mL of 1% lidocaine was administered as a test dose. The patient was observed for 10–15 min after test dose injection and asked for any signs of newly developed motor and sensory blocks. No additional drugs were administered if the patient complained of possible signs of intrathecal, intravascular, loculation or subdural injections such as severe paresthesia, pain, weakness, numbness or paralysis. Then, 10 mL of 0.9% NaCl was slowly injected and after injection of normal saline, 0.125% bupivacaine mixed with 5 mg dexamethasone was injected. After 5 min, 10 mL of 10% hypertonic saline was slowly injected under real-time fluoroscopic guidance while frequently asking the patient to describe any newly developed symptoms. The catheter was removed slowly to prevent catheter sheering, and the insertion site was sutured. The patient was moved to the postprocedure recovery unit in the supine position, and vital signs were continuously checked during recovery.

### 2.2. Outcome Measures

**Figure 2:**
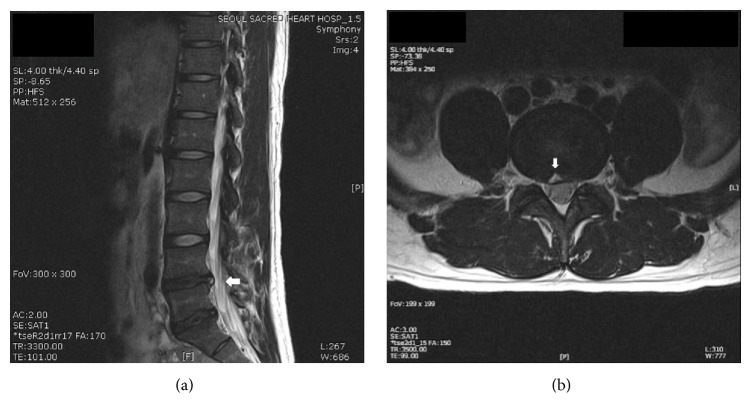
A T2-weighted magnetic resonance image showing the high-intensity zone (arrow) in a patient with a L4-5 herniated lumbar intervertebral disc. Sagittal view (a) and axial view (b).

The patient characteristics, outcome measures, and follow-up data were obtained and reviewed retrospectively from the computerized patient record system. Demographic data including age, gender, height, weight, and body mass index were collected. Past medical history including the presence of oral analgesic medication, history of physical therapies, history of blocks other than epidurals, and history of previous epidural injections were collected. Procedure-related clinical data and radiographic data included the type of herniated intervertebral disc (protrusion, extrusion, sequestration, and foraminal involvement), the presence of HIZ (Figure 2), and the level and location of the lesion. The terms disc protrusion and extrusion were defined according to the classification by Jensen et al. [20]. In this classification, the foraminal type of disc herniation occurs when the herniated intervertebral disc extends to and involves the vertebral foramen; this is type C in the Michigan State University (MSU) classification or the foraminal zone in the McCulloch classification [21, 22]. To evaluate the degree of pain relief after the procedure, the records of pain scores on the 11-point numerical rating scale (NRS: 0 = no pain; 10 = worst unbearable pain) before, 1 month after, and 12 months after the percutaneous epidural adhesiolysis procedure were collected. The patient satisfaction scores presented in a 7-scale global perceived effect scale (GPES: 7 = best ever; 6 = much improved; 5 = improved; 4 = not improved but not worse; 3 = worse; 2 = much worse; 1 = worst ever) which was checked 12 months after the percutaneous epidural adhesiolysis procedure were collected [23]. Responder analysis was performed with the definition of substantial response being a decrease ≥50% or an NRS score of 4 score compared to baseline without increasing the dose of oral medication [23, 24]. Information on any changes in the oral medication regimen at 1 month after the procedure was collected and classified as no change, decreased, or increased. The final epidurogram after the procedure was saved and the presence of transforaminal dye spread was also evaluated. Complications of vasculogram or myelogram during the procedure were noted, and immediate postprocedure complications were evaluated. Patients who needed repeated procedures during the first 12 months after percutaneous epidural adhesiolysis or surgery within the 12-month follow-up period were also identified.

The primary outcome was the factors associated with patients who had a favorable outcome 12 months after percutaneous epidural adhesiolysis treatment. The favorable outcome was defined as patients presenting substantial responses in the NRS pain score. Secondary outcomes included mean NRS scores at each time point, the proportion of substantial responders at each time point, complications, and the proportion of patients who needed surgical treatment during the 12-month follow-up period.

### 2.3. Statistical Analysis

Descriptive statistics were used to report the demographic and clinical characteristics. Continuous variables were presented as the mean with standard deviation (SD) or median with interquartile range (IQR). Categorical variables were expressed as absolute numbers with frequencies or percentages. For analysis of primary outcome, univariate analysis was first performed. The numerical variables were tested with Student's *t*-tests or the Mann-Whitney *U* test as appropriate, and categorical variables were tested using the chi-square test with Yate's correction or Fisher's exact test as appropriate. The odds ratio (OR) and 95% confidence interval (CI) were provided to show the reliability of the estimates. Multiple logistic regression analysis was used for the multivariate analysis to identify individual predictors of substantial response that were suggested by the univariate analysis. All *P* values < 0.05 by univariate analysis were included in the multivariate analysis. Continuous variables were first assessed for normality using the Shapiro–Wilk test. *P* values less than 0.05 were considered statistically significant, and two-tailed tests were used for all experimental outcomes. SAS version 9.3 (SAS Institute Inc., Cary, NC) was used for statistical analysis.

## 3. Results

A total of 875 lower back pain with radicular pain cases treated with percutaneous epidural adhesiolysis between November 2011 and October 2013 were collected and reviewed. Among these, 427 cases were diagnosed with lumbar disc herniation, of which 20 cases were removed from the analysis due to insufficient records. Thus, a total of 407 patients were analyzed. The demographic and clinical characteristics of the study patients are summarized in Table 1. The number of patients presenting with a substantial response 12 months after percutaneous epidural adhesiolysis treatment was 294 (72.2%), and the number of nonresponders was 113 (27.8%). The demographics, clinical characteristics, concurrent medications, initial treatment outcomes of percutaneous epidural adhesiolysis, and presence and type of complications during and after the procedure were compared between the substantial responders and nonresponders (Table 2). There was a statistically significant difference between the two groups regarding the prevalence of type of disc herniation (*P* = 0.005), initial NRS score at 1 month (*P* = 0.007), and the presence of HIZ (*P* = 0.002). The patients in the substantial responder group were more likely to have a lower proportion of disc herniation involving the vertebral foramen, have a HIZ on MRI, and have a lower pain score 1 month after percutaneous epidural adhesiolysis compared to the nonresponder group.

**Figure 3:**
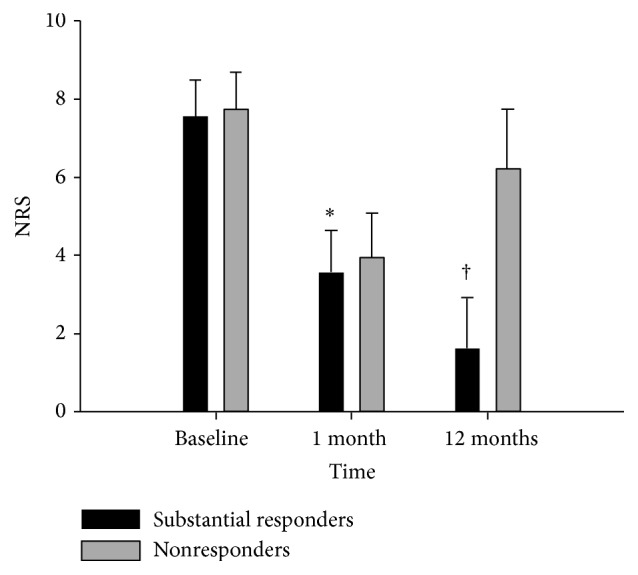
Change in numerical rating scale (NRS) pain scores between the substantial responder and nonresponder groups during follow-up; ^*∗*^*P* = 0.007, ^†^*P* < 0.001.

**Figure 4:**
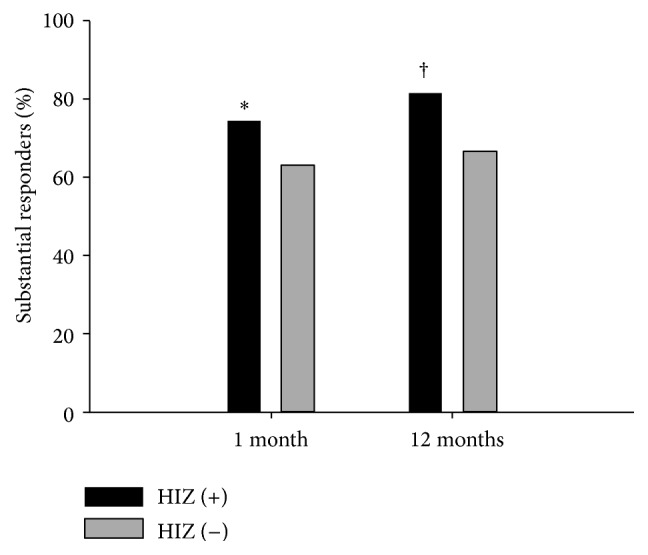
The proportion of substantial responders in the high-intensity zone (HIZ) positive patients and the HIZ negative patients; ^*∗*^*P* = 0.028, ^†^*P* = 0.002.

The results of univariate and multivariate analyses are listed in Table 3. Multivariate logistic regression analysis demonstrated that HIZ on MRI and a lower NRS score at 1 month were predictors of a successful response to percutaneous epidural adhesiolysis after 12 months, whereas the presence of lumbar disc herniation involving the vertebral foramen predicted an unsuccessful response after 12 months. The NRS pain scores in the responder and nonresponder groups are shown in Figure 3. The proportion of substantial responders of the HIZ positive and HIZ negative patients are shown in Figure 4. The number and proportion of patients demonstrating transforaminal dye spread to the affected nerve root in the final epidurogram immediately after percutaneous epidural adhesiolysis was 156 (98.7%) in the HIZ positive patient group and 238 (95.6%) in the HIZ negative patient group, respectively (*P* = 0.141). Patient satisfaction with the treatment results was significantly different between the two groups. The substantial responder group had a mean GPES score of 5.69 (1.13), whereas the nonresponder group had a mean score of 2.19 (1.08) (*P* < 0.001). The total number of immediate complications was 26 (6.4%, Table 4), with no effect on the primary outcomes. The number of patients who required surgical treatment within the follow-up period of 12 months was 21 (5.2%).

## 4. Discussion

The results of our present study demonstrate that the presence of HIZ on MRI is an independent predictor of favorable long-term outcome in patients who have received percutaneous epidural adhesiolysis for the treatment of lower back pain with radicular pain that is caused due to lumbar disc herniation. In contrast, vertebral foraminal involvement by the herniated lumbar disc was a predictor of unsuccessful outcome after percutaneous epidural adhesiolysis.

Symptomatic lumbar disc herniation with radiculopathy accompanies lower back pain in approximately 10% of cases [3]. The lifetime prevalence of lower back pain with radiculopathy is about 1.6% [25], but between 45 and 64 years of age, the prevalence increases to 23.7% [3]. In 90% of these patients, symptoms improve within 12 weeks [26], but some studies have shown that more than 30% of patients have some degree of pain and disability even after 1 year [6]. The initial treatment is conservative management, as the majority of patients are expected to have symptom relief in a couple of weeks. However, in cases of severe symptoms that can severely affect daily life or if symptoms persist for more than several weeks, procedures such as epidural steroid injections can be employed. There is sufficient evidence that epidural steroid injections give short-term pain relief [27], but its ability to decrease the rate of surgery is controversial [28–30]. In addition, the success rate of epidural steroid injections for the treatment of radiculopathy due to lumbar disc herniation varies between studies, ranging from 42% to 77% [31–34] and the rate of surgical procedures after epidural steroid injection was between 10% and 25% [3, 28]. To date, there have been few studies on percutaneous epidural adhesiolysis for the treatment of lower back pain with radiculopathy caused by lumbar disc herniation. One prospective study compared percutaneous epidural adhesiolysis with placebo for the treatment of chronic lumbar radicular pain caused by lumbar disc herniation or failed back surgery. The results of percutaneous epidural adhesiolysis were significantly more superior compared with placebo, but the pathology of patients in that study was heterogenic [9]. The results of our current study showed a 1-year response rate of 72.2%, and the failure rate (proportion of patients who needed surgery) was 5.2%, which is in accordance with data from previous studies on percutaneous epidural adhesiolysis and epidural steroid injections [9, 32, 33].

In our present study, we further evaluated the prognostic factors for the successful treatment of percutaneous epidural adhesiolysis. The presence of HIZ and foraminal involvement of the lesion were the 2 important prognostic factors for successful treatment response. The HIZ on the T2-weighted MRI was first described by Aprill and Bogduk in 1992 [35]. It is currently known that HIZ reflects painful tears of the annulus fibrosus in the intervertebral disc. However, the correlation between the presence of HIZ on MRI and the severity of symptoms are known to be controversial [36]. Previous studies have reported high specificity and a positive predictive value of HIZ, but the results of sensitivity varied from 26.7% to 81% [35, 37–39]. The prevalence of HIZ in lower back pain patients is between 25% and 59% [40, 41], but it is also found in 6–33% of asymptomatic patients [42, 43]. The proportion of patients who presented with HIZ in our current study was 38.8%, and interestingly, these patients had a significantly higher rate of treatment success compared to patients without an HIZ (81% versus 66.7%). Previous pathological studies have demonstrated evidence that tissues at the site of the HIZ are disorganized, vascularized, and granulated with significant numbers of inflammatory cells [44, 45]. These reports support the notion that location of the HIZ may be the main lesion of inflammation that causes pain in symptomatic patients, thus making it the direct target of delivery of local anesthetics and steroids by percutaneous epidural adhesiolysis, which may contribute to the superior outcome. Another possible hypothesis is the role of hypertonic saline. Annular tears are filled with fluid or mucoid materials, and injection of hypertonic saline may promote the absorption of fluids and mucoid. Previous studies have demonstrated the efficacy of adding hypertonic saline during transforaminal injections and epidural adhesiolysis [46, 47]. Hypertonic saline is also known to have inhibitory effect on fibroblast cell proliferation and it is thought to have neuromodulatory effects according to previous experimental studies. The high concentrated chloride ions are known to promote persistent C fiber blockade and contribute to changes in pain conductivity [48, 49]. However, administration of hypertonic saline is known to have several complications, thus special care must be taken during the injection of hypertonic saline. The possible complications are pain during injection, paresthesia, and chemical arachnoiditis. Furthermore, the spread of hypertonic saline in the diseased epidural space or to the subdural space is difficult to predict, due to scarring, narrowing, and adhesion. To clarify the role of steroids and hypertonic saline on HIZ, further prospective studies that compare and quantify the extent of HIZ through radiologic images before and after treatment seem essential.

Previous studies that have evaluated the outcome of percutaneous epidural adhesiolysis reported foraminal stenosis as a predictor of unsuccessful treatment outcome [16, 17]. This is due to the difficulty of delivering the desired medication to the target lesion. Furthermore, when caudal epidural steroid injections have been performed, patients presenting with lumbar herniated discs involving the foraminal zone demonstrated significantly unfavorable treatment outcomes compared to those with discs herniated in the central zone [50]. For the treatment of lesions involving the foramen, utilization of steerable catheter devices may provide a better outcome in this group of patients [10, 51].

There were several noteworthy limitations of our present study. First, only patients who had sufficient data and who were followed up for 12 months were included in our analysis, and those with inadequate data or who were lost to follow-up were excluded. Although the number of follow-up losses or inadequate records was relatively small, and did not exceed 5% of the total number of cases, there is a possibility that these losses may have been due to dissatisfaction with the treatment results. Indeed, in prospective studies with an intention-to-treat design, follow-up losses are occasionally considered to be treatment failures [47]. Second, we did not assess the functional, emotional, and quality of life index, which is an important outcome domain in chronic pain studies [52], because the data were analyzed based on preexisting medical records, which is a limitation of retrospective studies. Third, the classification of lumbar disc herniation is currently not standardized [53]; thus, different interpretations of the results are possible. In this study, we used the classification developed by Jensen et al. [20] and added foraminal type to the MSU and McCulloch classification [21, 22].

In conclusion, the presence of HIZ on MRI is likely to predict a favorable outcome after percutaneous epidural adhesiolysis treatment in patients diagnosed with lumbar disc herniation who present lower back pain with radicular pain. The presence of foraminal disease is a prognostic indicator of an unfavorable outcome. These results may expand the indications for percutaneous epidural adhesiolysis but should be validated by a future randomized prospective study.

## Figures and Tables

**Table 1:** Demographic and clinical characteristics of the study patients (*n* = 407).

Variables		Values
Gender	Male	173 (42.5%)
	Female	234 (57.5%)

Age		52.00 (14.15)

Height		164.32 (8.32)

Weight		65.65 (10.62)

BMI		24.21 (2.55)

Type of HIVD	Protrusion	167 (41.0%)
	Extrusion	175 (43.0%)
	Sequestration	45 (11.1%)
	Foraminal	20 (4.9%)

Concurrent oral analgesics	Yes	400 (98.3%)
	No	7 (1.7%)

Concurrent physical therapy	Yes	190 (46.7%)
	No	217 (53.3%)

Previous epidural block	Yes	142 (34.9%)
	No	265 (65.1%)

Previous blocks other than epidural	Yes	16 (3.9%)
	No	391 (96.1%)

Number of levels	Single	254 (62.4%)
	Two	126 (31.0%)
	Three	27 (6.6%)

Location of lesion	Left	190 (46.7%)
	Right	182 (44.7%)
	Both	35 (8.6%)

HIZ at MRI	Present	158 (38.8%)
	Negative	249 (61.2%)

Baseline NRS score		7.595 (0.93)

The values are presented as a mean (SD) or absolute number (percentage).

BMI: body mass index, HIVD: herniated intervertebral disc, HIZ: high-intensity zone, MRI: magnetic resonance image, NRS: numerical rating scale, SD: standard deviation.

**Table 2:** Results for substantial responders compared to nonresponders 12 months after percutaneous epidural adhesiolysis, as classified by demographic and clinical variables.

Variables		Values		*P*
		Substantial responders (*N* = 294)	Nonresponders (*N* = 113)	
Gender	Male	131 (44.6%)	42 (37.2%)	0.215
	Female	163 (55.4%)	71 (62.8%)	

Age		51.5 (14.1)	53.3 (14.2)	0.175

Height		164.5 (8.4)	163.8 (8.2)	0.404

Weight		65.7 (10.9)	65.4 (9.8)	0.832

BMI		24.2 (2.6)	24.3 (2.4)	0.526

Type of HIVD	Protrusion	120 (40.8%)	47 (41.6%)	0.005
	Extrusion	135 (45.9%)	40 (35.4%)	
	Sequestration	31 (10.6%)	14 (12.4%)	
	Foraminal	8 (2.7%)	12 (10.6%)	

Concurrent oral analgesics	Yes	288 (98.0%)	112 (99.1%)	0.706
	No	6 2.0%)	1 (0.9%)	

Concurrent physical therapy	Yes	130 (44.2%)	60 (53.1%)	0.134
	No	164 (55.8%)	53 (46.9%)	

Previous epidural block	Yes	98 (33.3%)	44 (38.9%)	0.344
	No	196 (66.7%)	69 (61.2%)	

Previous blocks other than epidural	Yes	9 (3.1%)	7 (6.2%)	0.241
	No	285 (96.9%)	106 (93.8%)	

Number of levels	Single	182 (61.9%)	72 (63.7%)	0.796
	Two	91 (31.0%)	35 (31.0%)	
	Three	21 (7.1%)	6 (5.3%)	

Location of lesion	Left	135 (45.9%)	55 (48.7%)	0.874
	Right	133 (45.2%)	49 (43.4%)	
	Both	26 (8.9%)	9 (7.9%)	

HIZ at MRI	Present	128 (43.5%)	30 (26.5%)	0.002
	Negative	166 (56.5%)	83 (73.5%)	

Baseline NRS score		7.55 (0.91)	7.71 (0.96)	0.139

NRS score at 1 month		3.56 (1.09)	3.94 (1.14)	0.007

Substantial response at 1 month	Yes	205 (69.7%)	69 (61.1%)	0.121
	No	89 (30.3%)	44 (38.9%)	

Oral analgesic change at 1 month	No change	253 (86.1%)	95 (84.1%)	0.772
	Decreased	40 (13.6%)	17 (15.0%)	
	Increased	1 (0.3%)	1 (0.9%)	

Repeated procedure	Yes	11 (3.8%)	4 (3.5%)	0.844
	No	283 (96.2%)	109 (96.5%)	

Vasculogram	Yes	19 (6.5%)	8 (7.1%)	0.999
	No	275 (93.5%)	105 (92.9%)	

Myelogram	Yes	10 (3.4%)	2 (1.8%)	0.586
	No	284 (96.6%)	111 (98.2%)	

Immediate complication	Yes	20 (6.8%)	6 (5.3%)	0.745
	No	274 (93.2%)	107 (94.7%)	

The data are presented as a mean (SD) or absolute number (percentage).

BMI: body mass index, HIVD: herniated intervertebral disc, HIZ: high-intensity zone, MRI: magnetic resonance image, NRS: numerical rating scale, SD: standard deviation.

**Table 3:** Univariate and multivariate analyses of variables that predict a substantial response 1 year after percutaneous epidural adhesiolysis.

		Univariate			Multivariate		
		OR	95% CI	*P*	OR	95% CI	*P*
Gender	Male	1					
	Female	0.736	0.469–1.145	0.178			

Age		0.991	0.975–1.006	0.237			

Height		1.010	0.984–1.037	0.466			

Weight		1.003	0.983–1.024	0.767			

BMI		0.985	0.904–1.073	0.727			

Type of HIVD	Protrusion	1					
	Extrusion	1.322	0.812–2.160	0.262	1.409	0.852–2.345	0.183
	Sequestration	0.867	0.430–1.814	0.696	0.982	0.474–2.110	0.962
	Foraminal	0.228	0.081–0.603	0.004	0.295	0.101–0.809	0.020

Concurrent oral analgesics	Yes	1					
	No	2.333	0.393–44.329	0.435			

Concurrent physical therapy	Yes	1					
	No	1.428	0.925–2.211	0.109			

Previous epidural block	Yes	1					
	No	1.275	0.811–1.995	0.289			

Previous blocks other than epidural	Yes	1					
	No	2.091	0.731–5.752	0.153			

Number of levels	Single						
	Two	1.029	0.642–1.667	0.908			
	Three	1.385	0.567–3.898	0.501			

Location of lesion	Left	1					
	Right	1.106	0.703–1.743	0.664			
	Both	1.177	0.534–2.805	0.697			

HIZ at MRI	Present	1					
	Negative	0.469	0.288–0.748	0.002	0.507	0.305–0.827	0.007

Immediate complications	Yes	1					
	No	0.768	0.275–1.860	0.582			

Baseline NRS score		0.833	0.657–1.053	0.126			

1-month NRS score		0.735	0.600–0.894	0.002	0.789	0.640–0.969	0.025

Oral analgesic consumption after 1 month	No change	1					
	Decreased	0.884	0.485–1.669	0.693			
	Increased	0.375	0.015–9.559	0.490			

Substantial response after 1 month	Yes	1					
	No	0.681	0.434–1.074	0.096			

Repeated procedure during follow-up	Yes	1					
	No	0.944	0.257–2.827	0.923			

BMI: body mass index, CI: confidence intervals, HIVD: herniated intervertebral disc, HIZ: high-intensity zone, MRI: magnetic resonance image, NRS: numerical rating scale, OR: odds ratio.

**Table 4:** Types of immediate postprocedure complications in the substantial responder and nonresponder groups.

Complication	Group	
	Responders (*N* = 20)	Nonresponders (*N* = 6)
Decreased metal status	6	1
Dizziness	1	
Hypotension	4	1
Motor weakness	2	1
Decreased sensory	3	
Chest pain	2	1
Postprocedure pain	3	2
Dyspnea	1	1
Nausea	1	

The data are presented as absolute numbers.

## Additional Points

*Summary*. In this study, we retrospectively evaluated the factors predicting the outcome of percutaneous epidural adhesiolysis on patients diagnosed with lumbar disc herniation by review of electronic medical records. Multivariate logistic regression analysis demonstrated that positive high-intensity zone (HIZ) at magnetic resonance imaging (MRI) was a predictor of successful substantial response to percutaneous epidural adhesiolysis for 12 months and the presence of a lumbar disc herniation involving the vertebral foramen was a predictor for unsuccessful response after 12 months.
